# OsBSK3 Positively Regulates Grain Length and Weight by Inhibiting the Phosphatase Activity of OsPPKL1

**DOI:** 10.3390/plants11121586

**Published:** 2022-06-16

**Authors:** Peng Tian, Jiafan Liu, Baohui Yan, Shuai Li, Bin Lei, Rongxin Shen, Cailin Lei, Miaoyun Xu

**Affiliations:** 1 Biotechnology Research Institute, Chinese Academy of Agriculture Sciences, Beijing 100081, China; tianpengtdc@163.com (P.T.); m17854269570@163.com (B.Y.); 2 College of Life Sciences, Shandong Agricultural University, Tai’an 271018, China; liujiafan2003@163.com; 3 National Key Facility for Crop Gene Resources and Genetic Improvement, Institute of Crop Science, Chinese Academy of Agriculture Sciences, Beijing 100081, China; lishuai19961221@163.com; 4 College of Agronomy, Hunan Agricultural University, Changsha 410128, China; leibin619@163.com; 5 State Key Laboratory for Conservation and Utilization of Subtropical Agro-Bioresources, College of Life Sciences, South China Agricultural University, Guangzhou 510642, China; shenrongxin@scau.edu.cn

**Keywords:** rice, grain size, brassinosteroids, *OsBSK3*

## Abstract

Brassinosteroids (BRs) are a crucial class of plant hormones that regulate many important agronomic traits in rice (*Oryza sativa* L.); thus, the BR signaling pathway is a very important tool for breeders to improve the grain yield and quantity of rice. Contrary to the well-established BR signaling pathway in *Arabidopsis*, there are significant gaps in the rice BR signaling pathway, especially the regulation mechanism from OsBSK3 to OsPPKLs and OsGSKs. In this study, we report how *OsBSK3* knockout mutants confer shorter and lighter grains and exhibit a typical BR-insensitive phenotype, suggesting *OsBSK3* plays a positive role in BR signaling without genetic redundancy with homologs. Furthermore, OsBSK3 could physically interact with OsPPKL1 and OsGSK3, the downstream components in BR signaling, as a scaffold protein, and inhibit the phosphatase activity of OsPPKL1 on the dephosphorylation of OsGSK3. In addition, the genetic evidence showed *OsBSK3* acts upstream of *OsPPKL1* in regulating grain length and weight. Our results clarify the role of *OsBSK3* and provide new insights into BR-signaling mechanisms, leading to potential new targets for the genetic improvement of rice.

## 1. Introduction

Rice (*Oryza sativa* L.) is an important food crop worldwide and increasing grain yield is a major goal of rice breeding. Grain size and shape (mainly specified by length, width and thickness) are major determining factors for grain weight and yield, and also affect the quality and market value of rice [1,2,3]. Therefore, in-depth research on the mechanisms of grain size and shape facilitates the breeding of high yield and quality rice.

Brassinosteroids (BRs) play important roles in regulating grain size and shape. In addition, BRs as major growth-promoting hormones play crucial roles in regulating various biological processes, including plant height, leaf angle, grain size and weight, suggesting that the manipulation of BR biosynthesis or signaling may provide promising strategies for improving rice yield [4,5]. BR-signaling mechanisms have been intensively studied in *Arabidopsis*. Briefly, BRs are perceived by the membrane receptor-like kinase BRASSINOSTEROID INSENSTIVE 1 (BRI1) and its co-receptor BRI1-ASSOCIATED RECEPTOR KINASE 1 (BAK1), and the signal is transduced to the central transcriptional factors BRASSINAZOLE RESISTANT 1 (BZR1) and bri1-EMS-SUPPRESSOR 1 (BES1/BZR2) through a series of phosphorylating or dephosphorylating events involving BR-SIGNALING KINASEs (BSKs), BRI1-SUPPRESSOR 1 (BSU1) and BRASSINOSTEORID INSENSITIVE 2 (BIN2). Among them, BSKs play central positive roles in controlling BR signaling [6,7,8,9]. In contrast, knowledge of BR signaling in rice is still rather limited. Some BR signaling core components orthologous to the known *Arabidopsis* genes, such as *OsBRI1*, *OsBAK1*, *OsGSK2* (the orthologs of *BIN2*) and *OsBZR1*, have been shown to play conserved roles of BR signaling in rice, suggesting that the BR-signaling pathway is conserved in rice and *Arabidopsis* [8,10,11,12,13,14].

However, some unique BR-signaling components have been identified in rice that display different regulatory mechanisms from those in *Arabidopsis*. For example, *GRAIN LENGTH 2* (*GL*2) encodes GROWTH-REGULATING FACTOR 4 (OsGRF4) and affects grain size and rice yield though BR signaling. The transcription activation activity of *OsGRF4* could be inhibited by OsGSK2, the central negative regulator of rice BR signaling [15]. *GW5* encodes a calmodulin-binding protein, which is a major QTL affecting grain width and yield. GW5 physically interacts with OsGSK2 and represses its kinase activity, resulting in the accumulation of dephosphorylated OsBZR1 and DLT proteins in the nucleus to mediate growth responses (including grain width and weight) [16]. The major grain-length QTL *qGL3* encodes a putative protein phosphatase with Kelch-like repeat domains (OsPPKL1), an ortholog of AtBSU1. However, OsPPKL1 plays as a negative role in BR signaling, in contrast to AtBSU1. qGL3^9311^ dephosphorylates and stabilizes OsGSK3, which suppresses BR signaling, resulting in decreased grain length [12]. Although many rice BR-signaling elements have been cloned successively, the regulatory mechanisms of BR signaling in rice have not been fully clarified. *OsBSK3*, the ortholog of *AtBSK3*, belongs to a sub-family of receptor-like cytoplasmic kinases (RLCKs) (RLCK-XII) and is reported to play a conserved role in regulating BR signaling [17]. OsBSK3 could be phosphorylated by OsBRI1, and the phosphorylated OsBSK3 increases the binding affinity for *Arabidopsis* bri1-SUPPRESSOR1 (AtBSU1). *OsBSK3* RNAi co-suppression lines (the expression of *OsBSK1~4* is suppressed) showed smaller and lighter seeds than wild type (WT) plants [17]. However, how *OsBSK3* regulates downstream genes, such as *OsPPKLs* and *OsGSKs*, remained unclear.

In this study, we found that OsBSK3 positively controls grain size and weight without genetic redundancy with its homologs. Furthermore, OsBSK3 could physically interact with OsPPKL1 and OsGSK3, the downstream components in BR signaling, and inhibit the phosphatase activity of OsPPKL1 on the dephosphorylation of OsGSK3. Moreover, we demonstrate *OsBSK3* acts upstream of *OsPPKL1* in regulating grain length and weight in genetics. Our results provide new insights into BR-signaling mechanisms and may help improve grain yield and quantity in rice.

## 2. Results

### 2.1. OsBSK3 Is Involved in BR Signaling to Regulate Both Plant Architecture and Grain Size in Rice

To investigate the function of the BSK family in rice, we made a phylogenetic analysis based on the full-length protein sequences of OsBSKs and their orthologous proteins in *Arabidopsis*. The phylogenetic tree revealed that OsBSK3 is evolutionarily related to AtBSK3, AtBSK4, AtBSK7 and AtBSK8, members that have been reported to be involved in BR signaling in *Arabidopsis* (Figure S1).

**Figure 1:**
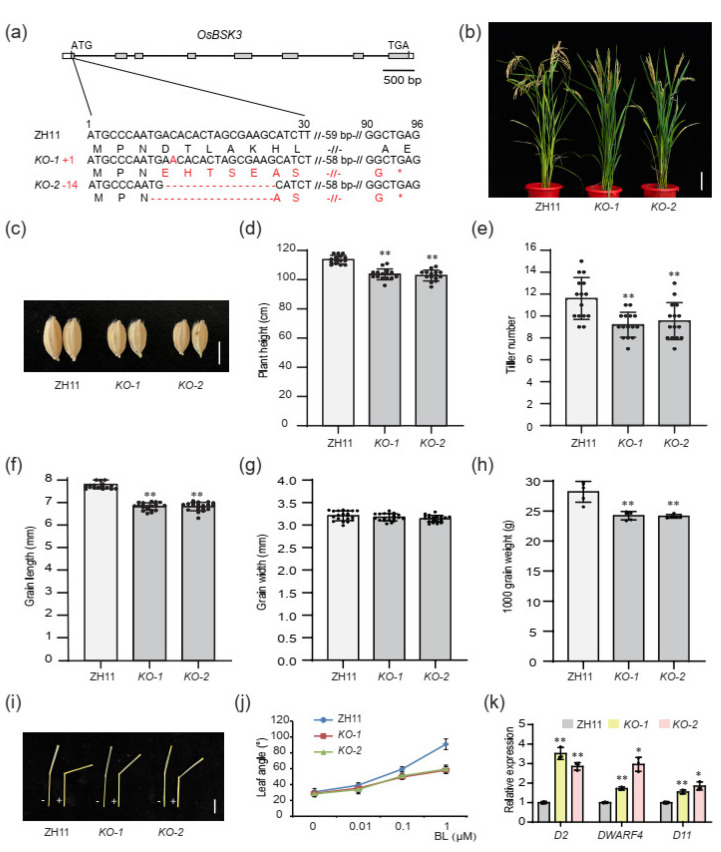
*OsBSK3* acts as a positive regulator in BR signaling. (**a**) Sequence analysis of the target sites in WT (ZH11) and *OsBSK3* knockout lines (*KO-1/-2*). In *KO-1* and *KO-2* mutants, a 1 bp insertion and a 14 bp deletion in the first exons results in different reading frame shifts and early termination in the second exon. (**b**,**c**) The gross morphology (**b**) and grain morphology (**c**) of ZH11 and *KO-1/-2*. Scale bars: 10 cm in (**b**); 0.5 cm in (**c**). (**d**–**h**) Quantification of plant height (**d**), tiller number (**e**), grain length (**f**), grain width (**g**) and 1000-g weight (**h**) of ZH11 and *KO-1/-2* lines. Values are means ± SD (*n* = 15 in (**d**) and (**e**); *n* = 20 in (**f**,**g**); *n* = 5 in (**h**)). (**i**) BL sensitivity test of ZH11 and *KO-1/-2* lines by lamina inclination experiments. The plus (+) and minus (−) respectively indicate treatments with and without BL (1 μM). Scale bar: 1 cm. (**j**) Lamina inclination assays of ZH11 and *KO-1/-2* in a range of BL concentrations. Bars indicate SD (*n* = 15). (**k**) qRT-PCR analysis of *D2, DWARF4* and *D11* in the flag leaves of ZH11 and *KO-1/-2* lines. Values are means ± SD (*n* = 3). * indicates significance at *p* < 0.05; ** indicates significance at *p* < 0.01 by two-tailed Student’s *t-*test.

To investigate the role of *OsBSK3*, we generated *OsBSK3* knockout plants using the CRISPR/Cas9 technology in the Zhonghua 11 (ZH11) background. Two independent lines (*KO-1* and *KO-2*) with a 1 bp insertion and a 14 bp deletion, respectively, in the first exon of *OsBSK3*, resulting in different reading frame shifts, were selected and used for phenotypic investigation (Figure 1a). Compared with the WT, both *KO-1* and *KO-2* plants exhibited typical BR-defective phenotypes, including lower plant height, lower tiller number, shortened grain length and reduced grain weight (Figure 1b–h).

In addition, the lamina inclination experiment showed that the seedlings of *KO-1* and *KO-2* were less sensitive to exogenous brassinolide (BL) treatment compared to the WT (Figure 1i,j). Correspondingly, the expression levels of three BR biosynthetic genes, *D2*, *DWARF4* and *D11*, which are usually upregulated in BR-deficient mutants through a feedback mechanism [18], were significantly elevated in the *KO-1* and *KO-2* plants (Figure 1k). These results indicated that *OsBSK3* is involved in BR signaling to regulate both plant architecture and grain size in rice without genetic redundancy with homologs.

### 2.2. OsBSK3 Physically Interacts with OsPPKL1 and OsGSK3

**Figure 2:**
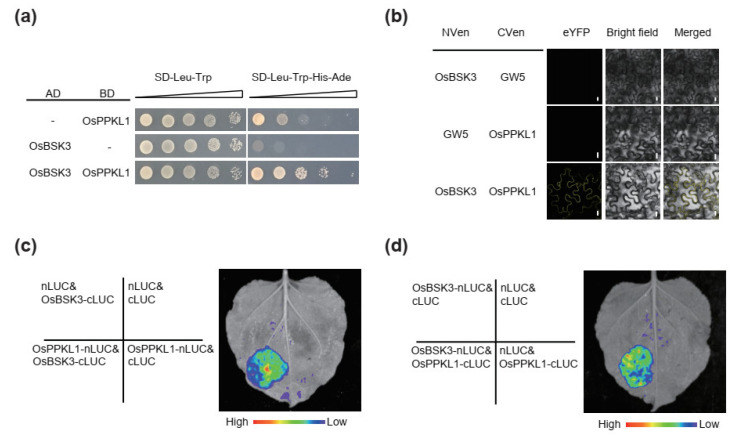
OsBSK3 interacts with OsPPKL1. (**a**) GAL4-based yeast two-hybrid (Y2H) assay showing the interaction between OsBSK3 and OsPPKL1. SD, synthetic dropout; AD, activation domain; BD, binding domain. The gradients indicate 10-fold serial dilutions. (**b**) BiFC analysis showing the interaction between OsBSK3 and OsPPKL1 in the epidermal cells of *N. benthamiana* leaves. NVen, N-terminal fragment of Venus fluorescent protein; CVen, C-terminal fragment of Venus fluorescent protein; GW5 is used as a negative control. Scale bars: 10 μm. (**c**,**d**) LCI assays to detect physical interaction between OsBSK3 and OsPPKL1 in the epidermal cells of *N. benthamiana* leaves. Colored scale bars indicate the luminescence intensity. nLUC, N terminus of LUC; cLUC, C terminus of LUC. Empty vectors are used as negative controls.

AtBSK3 was previously shown to form a complex with AtBSU1 and AtBIN2 as a scaffold protein in *Arabidopsis* [19]. OsPPKL1 (the ortholog of BSU1) was reported to interact with OsGSK3 (the ortholog of BIN2) and dephosphorylate it to modulate BR signaling in rice [12]. Due to the conserved function of *OsBSK3* in BR signaling between *Arabidopsis* and rice, we hypothesized that OsBSK3 may also form a complex with OsPPKL1 and OsGSK3 as a scaffold protein in rice. To explore this hypothesis, we first performed a yeast two-hybrid (Y2H) assay to test the interaction between OsBSK3 and OsPPKL1. The result showed that the OsBSK3 protein could strongly interact with OsPPKL1 (Figure 2a). Bimolecular fluorescence complementation (BiFC) analysis was further used to determine whether OsBSK3 interacts with OsPPKL1. The known GW5 was utilized as a negative control. The epidermal cells of *Nicotiana benthamiana* leaf co-expressing OsBSK3 fused to the N-terminal half of yellow fluorescent protein Venus (NVen) and OsPPKL1 fused to the C-terminal half of Venus (CVen) showed a strong fluorescent signal, indicating that OsBSK3 interacted with OsPPKL1 (Figure 2b). Moreover, luciferase complementation imaging (LCI) assays also showed that the combination of OsBSK3 and OsPPKL1 could reconstitute the functional LUC activity, regardless of whether each was fused to the N- or C-terminus of LUC, while the negative controls could not (Figure 2c,d).

**Figure 3:**
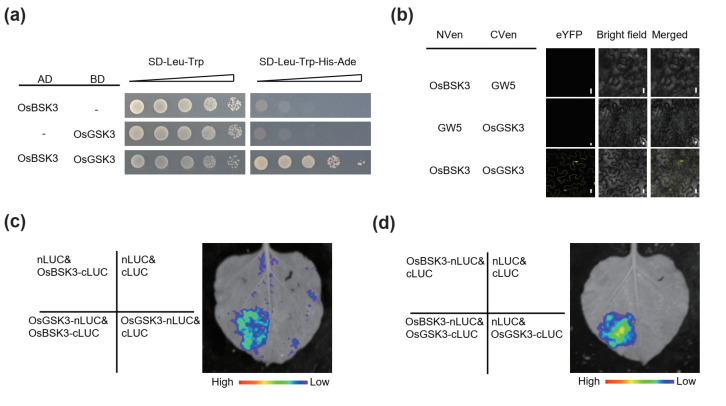
OsBSK3 interacts with OsGSK3. (**a**) GAL4-based yeast two-hybrid (Y2H) assay showing the interaction between OsBSK3 and OsGSK3. SD, synthetic dropout; AD, activation domain; BD, binding domain. The gradients indicate 10-fold serial dilutions. (**b**) BiFC analysis showing the interaction between OsBSK3 and OsGSK3 in the epidermal cells of *N. benthamiana* leaves. NVen, N-terminal fragment of Venus fluorescent protein; CVen, C-terminal fragment of Venus fluorescent protein; GW5 is used as a negative control. Scale bars: 10 μm. (**c**,**d**) LCI assays to detect physical interaction between OsBSK3 and OsGSK3 in the epidermal cells of *N. benthamiana* leaves. Colored scale bars indicate the luminescence intensity. nLUC, N terminus of LUC; cLUC, C terminus of LUC. Empty vectors are used as negative controls.

At the same time, we also examined the interaction between OsBSK3 and OsGSK3. The results of the Y2H, BiFC and LCI assays showed OsBSK3 interacts with OsGSK3 (Figure 3a–d). Taken together, our results suggest that OsBSK3 interacts with both OsPPKL1 and OsGSK3 and may form a complex with them.

### 2.3. OsBSK3 Inhibits the Phosphatase Activity of OsPPKL1

**Figure 4:**
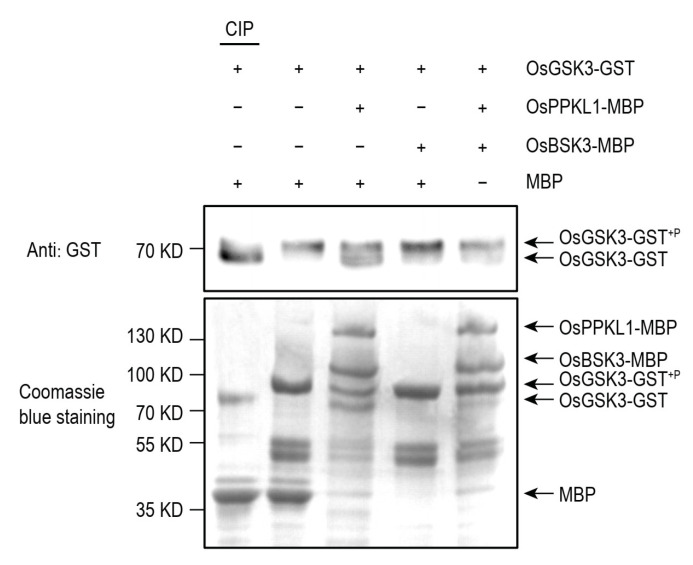
OsBSK3 inhibits the phosphatase activity of OsPPKL1. *In vitro* autophosphorylation assay of OsGSK3-GST using a Phos-tag gel. The plus (+) and minus (−) respectively indicate addition and absence of the relevant protein. Proteins are detected by immunoblotting assays with anti-GST antibody. The lower panels show Coomassie blue staining of the proteins used for the phosphorylation assay.

OsPPKL1 was reported to dephosphorylate and stabilize OsGSK3 in rice [12]. As a scaffold with OsPPKL1 and OsGSK3, OsBSK3 may interfere with the phosphatase activity of OsPPKL1 on OsGSK3. Hence, we made the *in vitro* autophosphorylation assay of OsGSK3. The result showed that the purified OsGSK3-GST protein could autophosphorylate *in vitro*, and the self-phosphorylation level was decreased after adding the OsPPKL1-MBP protein, which was consistent with the description of previous reports [12]. Furthermore, the phosphatase activity of OsPPKL1-MBP on OsGSK3-GST was inhibited by OsBSK3-MBP, but not by MBP alone (Figure 4). These observations suggested that OsBSK3 could inhibit the phosphatase activity of OsPPKL1, thus promoting BR signaling in rice.

### 2.4. OsBSK3 Genetically Acts Upstream of OsPPKL1 in Regulating Grain Size

**Figure 5:**
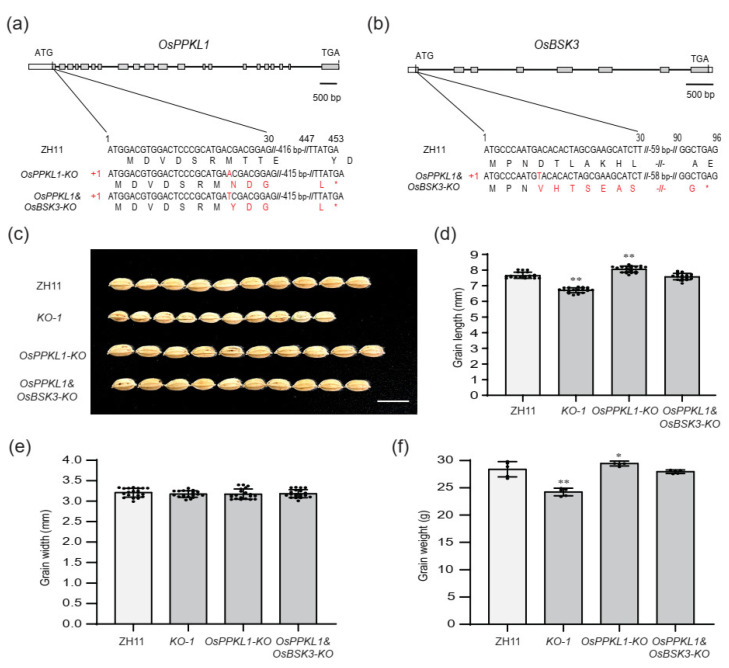
*OsBSK3* genetically acts upstream of *OsPPKL1* in regulating grain size. (**a**) Sequence analysis of *OsPPKL1* target sites in ZH11, *OsPPKL1-KO* and *OsPPKL1&OsBSK3-KO*. In these two mutants, 1 bp insertions in the first exons result in reading frame shifts and early termination, respectively. (**b**) Sequence analysis of *OsBSK3* target sites in ZH11 and *OsPPKL1&OsBSK3-KO*. In this mutant line, a 1 bp insertion in the first exon results in reading frame shift and early termination. (**c**) The grain morphology of ZH11, *OsBSK3* knockout line (*KO-1*), *OsPPKL1-KO* line and *OsPPKL1&OsBSK3-KO* line. Scale bar: 1 cm. (**d**–**f**) Quantification of grain length (**d**), grain width (**e**) and 1000-grain weight (**f**) of ZH11, *KO-1*, *OsPPKL1-KO* and *OsPPKL1&OsBSK3-KO* lines. Values are means ± SD (*n* = 20 in (**d**,**e**); *n* = 5 in (**f**)). * indicates significance at *p* < 0.05; ** indicates significance at *p* < 0.01 by two-tailed Student’s *t-*test.

To further examine the genetic relationship of *OsBSK3* with *OsPPKL1,* we knocked out *OsBSK3* and *OsPPKL1* in the ZH11 background using CRISPR/Cas9 technology, and obtained a homozygous *Osppkl1* mutant and a homozygous *OsPPKL1* and *OsBSK3* double-mutant plants, respectively, in T_0_. There were 1 bp insertions in the same site of the first exon of *OsPPKL1* both in the *OsPPKL1* mutant (*OsPPKL1-KO*) and the *OsPPKL1&OsBSK3* (*OsPPKL1&OsBSK3-KO*) double mutant, resulting in different reading frame shifts and early termination, respectively (Figure 5a). There was also a 1 bp insertion in the same site as *KO-1* in *OsBSK3* of the *OsPPKL1&OsBSK3-KO* mutant plant, resulting in different reading frame shifts and early termination (Figure 5b).

For comparing the grain phenotypes, the *osppkl1* mutant exhibited longer and heavier grains than the WT (Figure 5c,d,f), which is consistent with the description of previous reports [12]. Moreover, the *osppkl1/osbsk3* double mutant rescued the shorter and lighter grains of the *osbsk3* mutant line (*KO-1*) and displayed phenotypes similar to the WT line (Figure 5c,d,f), indicating that *OsBSK3* may act upstream of *OsPPKL1* in regulating grain size and weight. However, more evidence is needed to prove the genetic relationship between *OsBSK3* and *OsPPKL1.*

## 3. Discussion

BRs are a classic of plant-specific steroid phytohormones, controlling major aspects of plant growth and development in both dicots and monocots. BSK family members have been shown to play crucial roles in regulating BR signaling, but the regulatory mechanisms are unclear. Many BSK members (AtBSK1–6, 8 and 11) have been reported to be involved in BR signaling in *Arabidopsis* [20,21]. However, the genetic redundancy makes it difficult to unravel the function of this family. There are fewer BSK family members in rice (only 5 members) than *Arabidopsis* (12 members) [22], which provides a good material for studying the function of this family. Nevertheless, only *OsBSK3* has been reported to promote BR signaling in rice, but the precise molecular mechanism is unknown [17]. In this study, we demonstrate that *OsBSK3* plays a positive role in BR signaling without genetic redundancy with its homologs. Therefore, the functional study of *OsBSK3* is of great significance for unraveling the regulatory mechanisms of BSK family in BR signaling and the genetic improvement of rice.

Despite important advances in the understanding of the functional mechanisms of BRs in both *Arabidopsis* and rice in recent years, the regulatory mechanisms of the BSK family remain largely unknown. The BSK family members lack the conserved regions for kinase activity, such as the conserved DFG motif and the classical GXGXXG motif (glycine-rich loop). Therefore, BSKs are regarded as a type of pseudo-kinases and may work as scaffolds, bridging multiple proteins to form complexes [19,23]. In *Arabidopsis,* AtBSK3 could be phosphorylated by AtBRI1 and promoted to interact with AtBSU1, which in turn inhibits the kinase activity of AtBIN2 through the dephosphorylation of AtBIN2 [19]. However, the regulatory mechanisms of the BSK family in rice are still unclear. In our study, we demonstrate that OsBSK3 could interact with OsPPKL1 and OsGSK3 as a scaffold protein (Figure 3 and Figure 4), which is consistent with its orthologs in *Arabidopsis*. One intriguing finding of this work is that OsBSK3 inhibited the phosphatase activity of OsPPKL1 on OsGSK3 (Figure 5), which is the first report and provides clues to reveal the precise molecular mechanisms of the BSK family in rice. Exploring the relationship between OsBSK3 and other elements downstream of BR signaling, such as OsPPKL2, OsPPKL3 and OsGSK2, will be an interesting research avenue that will require future studies.

**Figure 6:**
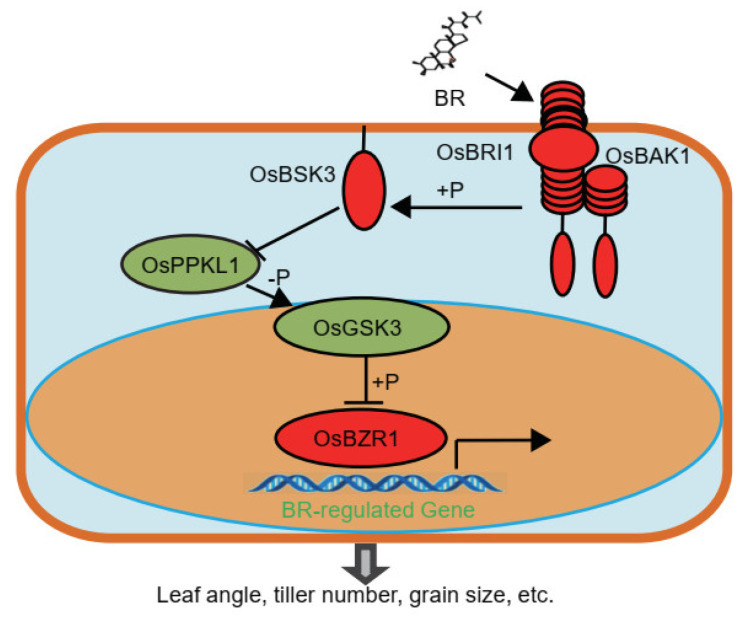
A model for the role of OsBSK3 in BR signaling. In this model, OsBSK3 could be phosphorylated by OsBRI1 and directly block the OsPPKL1’s phosphatase activity on OsGSK3, thus activating BR-regulated gene expression and triggering BR-mediated growth responses.

Compared with the elaborate BR-signaling mechanisms discovered in *Arabidopsis*, there are still significant gaps in our understanding of the BR-signaling pathway in rice [7,8,24]. In our case, we found that OsBSK3 inhibits the phosphatase activity of OsPPKL1 and genetically acts upstream of OsPPKL1 in BR signaling. Based on our findings, we propose a model to illustrate how OsBSK3 acts with downstream elements in BR signaling in rice (Figure 6). In this model, OsBSK3 could associate with OsPPKL1 and OsGSK3 as a scaffold protein. OsBSK3 inhibits the phosphatase activity of OsPPKL1 on OsGSK3 and triggers BR-mediated growth responses. This model fills in the gap of BR signaling in rice and provides new insights into BR signaling in general for understanding BR signaling in other crops.

Collectively, our study provides a novel mechanism in the regulation of BR signaling in rice, which differs from that in *Arabidopsis*. Our data support the positive regulatory role of *OsBSK3* in modulating grain length and weight though BR signaling, which will favor BR applications in molecular design for crop improvement.

## 4. Materials and Methods

### 4.1. Plant Materials and Growing Conditions

*Oryza sativa* cv. *Japonica* cultivar Zhonghua 11 (ZH11) was used for all transgenic experiments. The plants were grown in a paddy field at Shunyi Experimental Station of the Chinese Academy of Agricultural Sciences in Beijing during the natural growing seasons from 2019 to 2021.

Tobacco (*Nicotiana benthamiana* L.) seeds were sown directly into soil and grown in a culture room at 26 °C (16 h light/8 h dark) for 1 month before being used.

### 4.2. Phylogenetic Analysis

All relative protein sequences were retrieved from (http://rice.plantbiology.msu.edu/ (accessed on 2 July 2021)) and NCBI (National Center for Biotechnology Information, https://www.ncbi.nlm.nih.gov/ (accessed on 2 July 2021)). Phylogenetic analysis was conducted by MEGA 7.0 using the Neighbor-Joining method with 1000 bootstrap replications based on the full-length amino acid sequences of BSKs homologs.

### 4.3. Total RNA Extraction and Expression Analysis

Total RNA was extracted from the flag leaf of plants, using Trizol reagent (procuct of Invitrogen, Carlsbad, CA, USA). cDNA was synthesized using a QuantiTect Reverse Transcription Kit (procuct of QIAGEN, Dusseldorf, Germany) and used as the template for quantitative real-time PCR (RT-qPCR) analysis. The rice *ubiquitin* (LOC_Os03g10170) was used as an internal control. RT-qPCR assays were performed with an Applied Biosystems Q3 real-time PCR system, using the SYBR premix Ex TaqTM kit (product of Takara, Kusatsu, Shiga, Japan), according to the manufacturer’s recommended protocol. Each experiment was conducted with three biological replicates.

### 4.4. Plasmid Construction and Transformation

To generate the CRISPR/Cas9 constructs for *OsBSK3* and *OsPPKL1*, specific sites for targeted mutagenesis were designed online (http://www.e-crisp.org/E-CRISPR/designcrispr.html (accessed on 20 May 2020)) [25]. The CRISPR/Cas9 constructs were generated as previously described [26]. All constructs were confirmed through DNA sequencing. Following this, these constructs were introduced into the *Agrobacterium tumefaciens* strain EHA105 and used to transform to the callus of ZH11. The mutant plants were genotyped using PCR amplification and DNA sequencing. All transgenic lines were analyzed using the stable T_2_ progenies, unless otherwise indicated.

### 4.5. BL Treatment Lamina Inclination Assays

The lamina inclination assays after BL treatment were performed as previously described [27]. In brief, the lamina of 10-day-old seedlings were treated with 0, 0.01, 0.1 and 1 μM of BL. Images were captured 4 days after treatment, and the angles of the lamina joint bending were measured using ImageJ software (http://www.imagej.net (accessed on 15 January 2022)).

### 4.6. Protein Interaction Assays

For GAL4-based yeast Y2H assays, the CDSs of *OsBSK3, OsPPKL1* and *OsGSK3* were cloned into the pGBKT7 at the *Eco*RI and *Bam*HI restriction sites or pGADT7 at the *Eco*RI and *Bam*HI restriction sites (procuct of Clontech, Mountain View, CA, USA). The interaction assay was performed according to the manufacturer’s instructions (Clontech Yeast Protocols Handbook). Both the bait and prey constructs were co-transformed into the yeast strain AH109 and grown on SD/-Leu/-Trp medium, then further selected on SD/-Leu/-Trp/-His/-Ade medium.

For the BiFC assays, the CDSs of *OsBSK3, OsPPKL1*, *OsGSK3* and *GW5* were cloned into the BiFC binary vector pDOE at the *Bam*HI or *Pml*I restriction sites, respectively [28]. The examined constructs were transformed into *N. benthamiana* leaves for BiFC assays as previously described [29]. The fluorescent signal was visualized at 48–72 h after infiltration by a Leica TCS-SP6 confocal microscope.

For the firefly luciferase complementation imaging (LCI) assays, the CDSs of *OsBSK3, OsPPKL1* and *OsGSK3* were respectively cloned into the pCAMBIA-NLuc or pCAMBIA-CLuc vectors at the *Sal*I and *Kpn*I restriction sites. All constructs were confirmed through DNA sequencing and were introduced into the *Agrobacterium tumefaciens* strain EHA105. *N. benthamiana* leaves were co-injected with different pairs of nLUC and cLUC constructs as previously described [29] and incubated at 26 °C for 48 h. To image the luciferase luminescence, the leaves were detached and sprayed with 20 mg/mL potassium luciferin (product of Gold Biotech, Saint Louis, MO, USA) and incubated in darkness for 5 min. The luciferase luminescence from the infiltrated area was imaged using Night Shade LB 985 system (product of Berthold, Bad Wildbad, Germany) with a 60 s exposure time, 4 × 4 binning, slow readout, and high gain. The quantification of luciferase activity was carried out with IndiGO software (version 2.03.0) using average luminescence counts per second.

### 4.7. In Vitro Phosphorylation and Phos-Tag Assays

The CDS of *OsBSK3* and *OsPPKL1* were cloned into the pMALc2x vector at the *Bam*HI restriction site. The CDS of *OsGSK3* was cloned into the pGEX-4T vector at the *Eco*RI restriction site. MBP- and GST-tagged recombinant proteins were purified according to the user’s manual, and were then incubated in kinase buffer (25 mM Tris-Hcl (pH 7.5), 10 mM MgCl_2_, 10 mM NaCl, 1.5 mM DTT, 100 μM ATP, 2× phosphatase inhibitor cocktail) at 30 °C for 1 h. Subsequently, the samples were analyzed using 10% SDS-polyacrylamide phos-tag gels as previously described [30]. The samples were then transferred to NC membranes and detected using the anti-GST antibody (New England Biolabs, Ipswich, MA, USA).

All of the primer sequences are listed in Table S1.

## Data Availability

Not applicable.

## Supplementary Materials

The following supporting information can be downloaded at: https://www.mdpi.com/article/10.3390/plants11121586/s1, Figure S1. Phylogenetic analysis of BSK family in *Arabidopsis* and rice; Table S1: Primers used in this study.
