# Supplementary material for: OsBSK3 Positively Regulates Grain Length and Weight by Inhibiting the Phosphatase Activity of OsPPKL1
Source: Plants (Basel). 2022 Jun 16;11(12):1586. doi: 10.3390/plants11121586 (PMC9229280; doi:10.3390/plants11121586)
Supplement: Supplementary file 1 [file plants-11-01586-s001.zip › plants-1766500-supplementary.pdf]

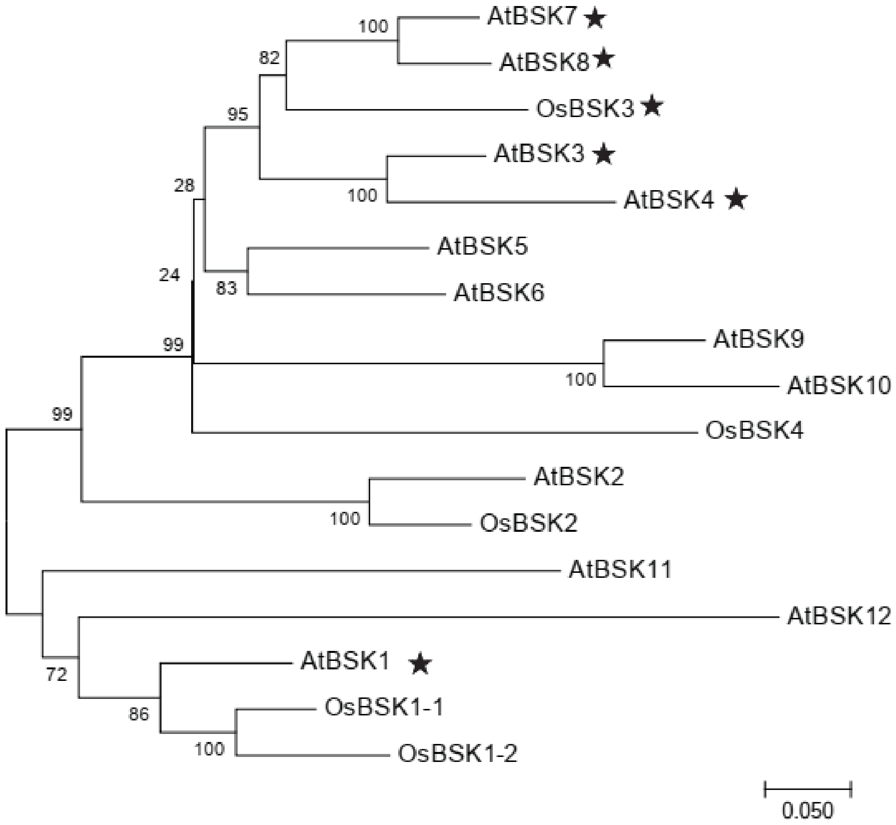

Figure S1. Phylogenetic analysis of BSK family in *Arabidopsis* and rice

Phylogenetic tree of BSK family in *Arabidopsis* and rice. Phylogenetic analysis was conducted using MEGA 7.0 with the full-length amino acid sequences of BSKs homologs. The bootstrap support is indicated above the branches. The black stars indicate the protein associated with BR signaling. The 0.050 scale represents the substitution distance.

Table S1. Primers used in this study

Primer name	Sequence 5’-3’	Purpose
qUBI-F	GCTCCGTGGCGGTATCAT	qPCR
qUBI-R	CGGCAGTTGACAGCCCTAG	qPCR
qD2-F	CCTTTTGGTGGTGGGCAGAG	qPCR
qD2-R	TGGGGAAGTTGACGATGTGGT	qPCR
qDWARF4-F	TGGGCTCTGAAACAATCTAACCT	qPCR
qDWARF4-R	CAAGGAAGAAGATGGCGAGG	qPCR
qD11-F	CAAGGGACAAGCAAGAAGTTTAC	qPCR
qD11-R	CGATTTCTATGGGCAGACCTC	qPCR
OsBSK3KO-gRT1	TGCTTCGCTAGTGTGTCATGTTTTAGAGCTAGAAAT	Transgenic construct
OsBSK3KO-U6bT1	ATGACACACTAGCGAAGCACAACACAAGCGGCAGC	Transgenic construct
OsPPKL1KO-gRT1	CGTGGACTCCCGCATGACGAGTTTTAGAGCTAGAAAT	Transgenic construct
OsPPKL1KO-U6aT1	TCGTCATGCGGGAGTCCACGCGGCAGCCAAGCCAGCA	Transgenic construct
OsBSK3ADEcoR1-F	CCATGGAGGCCAGTGAATTCATGCCCAATGACACACTAGCGA	Yeast Two-Hybrid assay
OsBSK3ADBamHI-R	CAGCTCGAGCTCGATGGATCCATTCAATGTCCACTGCTGCT	Yeast Two-Hybrid assay
OsPPKL1ADEcoR1-F	CCATGGAGGCCAGTGAATTCATGGACGTGGACTCCCGCATGACGA	Yeast Two-Hybrid assay
OsPPKL1ADBamHI-R	CAGCTCGAGCTCGATGGATCCTATCCAGGCAAGAGAACCTCGA	Yeast Two-Hybrid assay
OsBSK3BDEcoR1-F	CATGGAGGCCGAATTCATGCCCAATGACACACTAGCGA	Yeast Two-Hybrid assay
OsBSK3BDBamHI-R	CATGGAGGCCGAATTCATGGGCGGGCGCGTGTCCAAG	Yeast Two-Hybrid assay
OsPPKL1BDEcoR1-F	CATGGAGGCCGAATTCATGGACGTGGACTCCCGCATGACGA	Yeast Two-Hybrid assay
OsPPKL1BDBamHI-R	GCAGGTCGACGGATCCTTATATCCAGGCAAGAGAACCTCGA	Yeast Two-Hybrid assay
OsGSK3BDEcoR1-F	CATGGAGGCCGAATTCATGGCCACGCTGCCGGGCGG	Yeast Two-Hybrid assay
OsGSK3BDBamHI-R	GCAGGTCGACGGATCCGGTCCGGCAGCAGGCAAGAAA	Yeast Two-Hybrid assay

OsBSK3MCSI-BamH1-F	AACTAGTGGAGGATCCATGGGCGGGCGCGTGTCCAAG	Bimolecular fluorescence complementation assay
OsBSK3MCSI-BamH1-R	ACCCACCTCCGGATCCATGTCCACTGCTGCTACTTG	Bimolecular fluorescence complementation assay
GW5MCS1-BamHI-F	AACTAGTGGAGGATCCATGGGCAAGGCGGCGCGGTG	Bimolecular fluorescence complementation assay
GW5MCS1-BamHI-R	ACCCACCTCCGGATCCCCACCTCCTCTGCAAGAACG	Bimolecular fluorescence complementation assay
OsBSK3MCSII-PmlI-F	CCTACGTAGTCACGTGATGGGCGGGCGCGTGTCCAAG	Bimolecular fluorescence complementation assay
OsBSK3MCSII-PmlI-R	CTCCGGACGTCACGTGATGTCCACTGCTGCTACTTG	Bimolecular fluorescence complementation assay
OsPPKL1MCSII-PmlI-F	CCTACGTAGTCACGTGATGGACGTGGACTCCCGCATGACGA	Bimolecular fluorescence complementation assay
OsPPKL1MCSII-PmlI-R	CTCCGGACGTCACGTGTATCCAGGCAAGAGAACCTCGA	Bimolecular fluorescence complementation assay
OsGSK3MCSII-PmlI-F	CCTACGTAGTCACGTGATGGCCACGCTGCCGGGCGG	Bimolecular fluorescence complementation assay
OsGSK3-PmlI-R	CTCCGGACGTCACGTGCCGAGCATGCTCTGGTA	Bimolecular fluorescence complementation assay
OsBSK3-NlucSalI -F	CGGGGGACGAGCTCGGTACCATGGGCGGGCGCGTGTCCAAG	Firefly luciferase complementation Imaging assay
OsBSK3-NlucKpnI-R	ACGAGATCTGGTCGACATGTCCACTGCTGCTACTTG	Firefly luciferase complementation Imaging assay
OsBSK3-ClucSalI -F	ACGCGTCCCGGGGCGGTACCATGGGCGGGCGCGTGTCCAAG	Firefly luciferase complementation Imaging assay
OsBSK3-ClucKpnI-R	AGCTCTGCAGGTCGACTCAATGTCCACTGCTGCTACTTG	Firefly luciferase complementation Imaging assay
OsPPKL1-NlucSalI -F	CGGGGGACGAGCTCGGTACCATGGACGTGGACTCCCGCATGACGA	Firefly luciferase complementation Imaging assay
OsPPKL1-NlucKpnI-R	ACGAGATCTGGTCGACTATCCAGGCAAGAGAACCTCGA	Firefly luciferase complementation Imaging assay
OsPPKL1-ClucSalI -F	ACGCGTCCCGGGGCGGTACCATGGACGTGGACTCCCGCATGACGA	Firefly luciferase complementation Imaging assay
OsPPKL1-ClucKpnI-R	AGCTCTGCAGGTCGACTTATATCCAGGCAAGAGAACCTCGA	Firefly luciferase complementation Imaging assay
OsGSK3-NlucSalI -F	CGGGGGACGAGCTCGGTACCATGGCCACGCTGCCGGGCGG	Firefly luciferase complementation Imaging assay
OsGSK3-NlucKpnI-R	ACGAGATCTGGTCGACCCGAGCATGCTCTGGTA	Firefly luciferase complementation Imaging assay
OsGSK3-ClucSalI -F	ACGCGTCCCGGGGCGGTACCATGGCCACGCTGCCGGGCGG	Firefly luciferase complementation Imaging assay
OsGSK3-ClucKpnI-R	AGCTCTGCAGGTCGACTCACCGAGCATGCTCTGGTA	Firefly luciferase complementation Imaging assay
OsBSK3pMALc2xBamHI-F	TTCAGAATTCGGATCCATGGGCGGGCGCGTGTCCAAG	in vitro kinase assay
OsBSK3pMALc2xBamHI-R	CGACTCTAGAGGATCCTCAATGTCCACTGCTGCTACTTG	in vitro kinase assay
OsPPKL1-pMALc2xBamHI-F	TTCAGAATTCGGATCCATGGACGTGGACTCCCGCATGACGA	in vitro kinase assay

OsPPKL1-pMALc2xBamHI-R	CGACTCTAGAGGATCCTTATATCCAGGCAAGAGAACCTCGA	in vitro kinase assay
OsGSK3pGEX4tEcoRI-F	TGGATCCCCGGAATTCATGGCCACGCTGCCGGGCGG	in vitro kinase assay
OsGSK3pGEX4tEcoR1-R	GTCGACCCGGGAATTCTCACCGAGCATGCTCTGGTA	in vitro kinase assay
OsBSK3-identification-F	TGCTTCGCTAGTGTGTCAT	Transgenic identification
OsBSK3-identification-R	AGAACAACTCTTAATCTCAT	Transgenic identification
OsPPKL1-identification-F	CGTGGACTCCCGCATGACGA	Transgenic identification
OsPPKL1-identification-R	CGAGACCTCGTCGTCGTCGT	Transgenic identification
